# Pathomechanisms in schwannoma development and progression

**DOI:** 10.1038/s41388-020-1374-5

**Published:** 2020-07-02

**Authors:** Dario-Lucas Helbing, Alexander Schulz, Helen Morrison

**Affiliations:** 1 Leibniz Institute on Aging, Fritz Lipmann Institute, 07745 Jena, Germany; 2 Institute of Molecular Cell Biology, Jena University Hospital, Friedrich Schiller University Jena, 07745 Jena, Germany; 3 MVZ Human Genetics, 99084 Erfurt, Germany

**Keywords:** Cancer microenvironment, Inflammation

## Abstract

Schwannomas are tumors of the peripheral nervous system, consisting of different cell types. These include tumorigenic Schwann cells, axons, macrophages, T cells, fibroblasts, blood vessels, and an extracellular matrix. All cell types involved constitute an intricate “tumor microenvironment” and play relevant roles in the development and progression of schwannomas. Although *Nf2* tumor suppressor gene-deficient Schwann cells are the primary tumorigenic element and principle focus of current research efforts, evidence is accumulating regarding the contributory roles of other cell types in schwannoma pathology. In this review, we aim to provide an overview of intra- and intercellular mechanisms contributing to schwannoma formation.

“Genes load the gun, environment pulls the trigger.”

-George A. Bray

## Schwannomas are heterogeneous tumors involving multiple cell types

Research on Neurofibromatosis type 2 (NF2) and schwannoma pathobiology in recent years has moved toward a better mechanistic understanding of the interplay and crosstalk between the different cell types within the tumor. Schwannomas derive from tumorigenic Schwann cells, caused by loss-of-function mutations of the *Nf2* tumor suppressor gene. In contrast to other Schwann cell-derived tumors, e.g., neurofibromas, that involve multiple fascicles of a nerve, schwannomas grow without the entrapment of axons other than those associated with the Schwann cells from which the tumor originated [1]. Apart from tumorigenic Schwann cells and axonal processes of nerve cells, macrophages [2], T cells [3], endothelial cells [4], and sometimes perivascular B cells can be observed in schwannomas [3]. These different cell types influence each other through a variety of paracrine and juxtracrine mechanisms, which are discussed in this review.

**Fig. 1:**
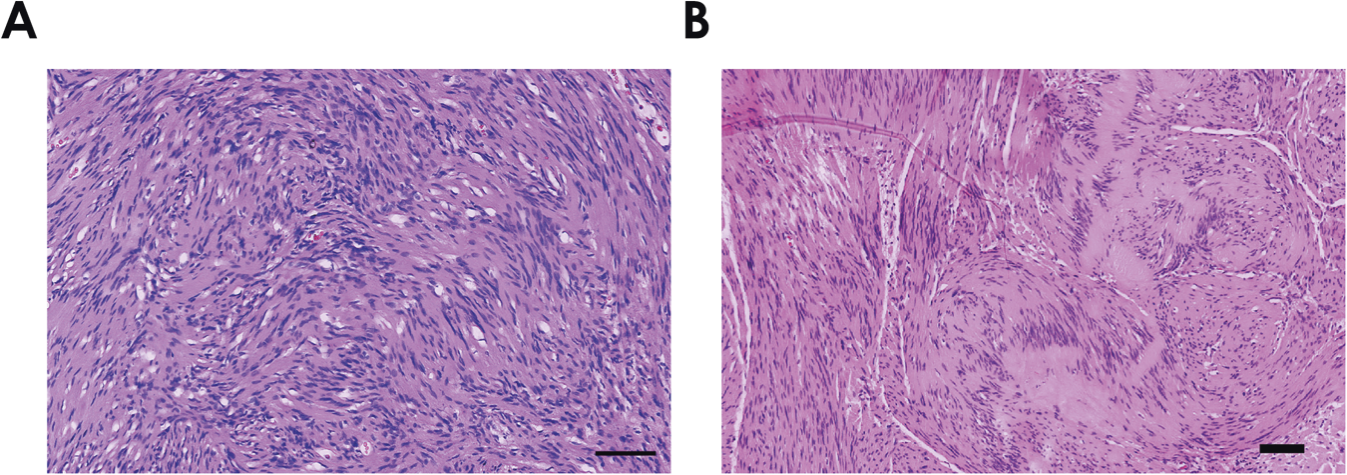
Histopathology of Antoni A tissue and Verocay bodies in schwannomas. **a** HE staining of a schwannoma. An Antoni A area with densely packed cells is shown. Most cell nuclei appear elongated, indicating the presence of tumor cells. Scale bar = 100 µm. **b** HE staining of a schwannoma. Zones lacking any nuclei alternate with areas of fusiform, fibrillary organized, elongated nuclei of tumor cell origin. These are typical Verocay bodies that are embedded into Antoni A tissue. Scale bar = 100 µm.

The Swedish neurologist Nils Ragnar Eugene Antoni was first to describe the heterogeneity of schwannomas by dividing the tumor tissue into two areas with distinct architectures. The first cell-rich Antoni type A tissue contains densely packed tumor cells, in which elongated nuclei form close palisades and alternating areas without any nuclei. These complexes of alternating tissue structures are called Verocay bodies (Fig. 1) [1].

Antoni A tissue may transform into Antoni type B tissue during tumor progression. Evidence for this hypothesis is provided by the observation of a “transition zone” at the borders between Antoni A and B tissue. Here, A-type tissue begins to degenerate and changes into B-type tissue. It has been shown that these transition zones display the highest proliferation indices compared with the pure A- or B-type areas, as well as a marked infiltration of phagocytic macrophages [5]. Macrophages are known to show high proliferation rates following activation [6] and are, together with Schwann cells, mainly responsible for the clearance of debris during peripheral nervous system (PNS) degeneration [7]. It is therefore reasonable to assume that macrophages contribute to the proliferative activity seen in the transition zone of schwannomas.

**Fig. 2:**
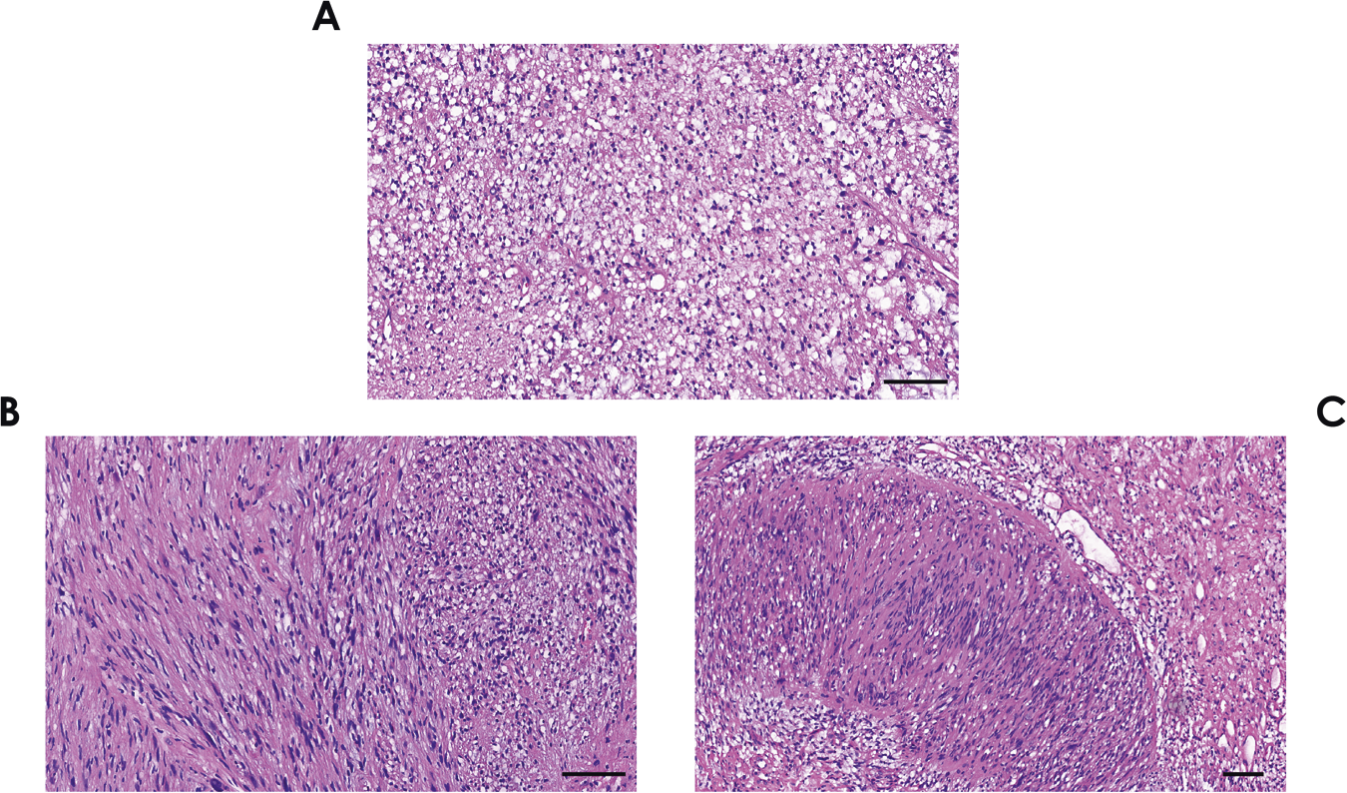
Histopathology of Antoni B tissue and transition zones in schwannomas. **a** HE staining of a schwannoma. All visible cells are loosely arranged and do not exhibit any particular organizational pattern. Various cell nuclei of different shape and size are shown, indicating the presence of various cell types. The stroma is loosened, the cellular organization exhibits a microcystic pattern. These vacuoles usually contain lipids. The presented tissue structure is typical for an Antoni B area. Scale bar = 100 µm. **b** HE staining of a schwannoma. A transition of Antoni A tissue (left; elongated, spindle-shaped nuclei) into Antoni B tissue with small and round nuclei and microcystic stroma (right) is depicted. Scale bar = 100 µm. **c** HE staining of a schwannoma. A transition of a Verocay body (in the center of the image) into Antoni B tissue is shown. The borders of the Verocay body merge into large vacuoles, indicating extensive tissue degradation. Scale bar = 100 µm.

**Fig. 3:**
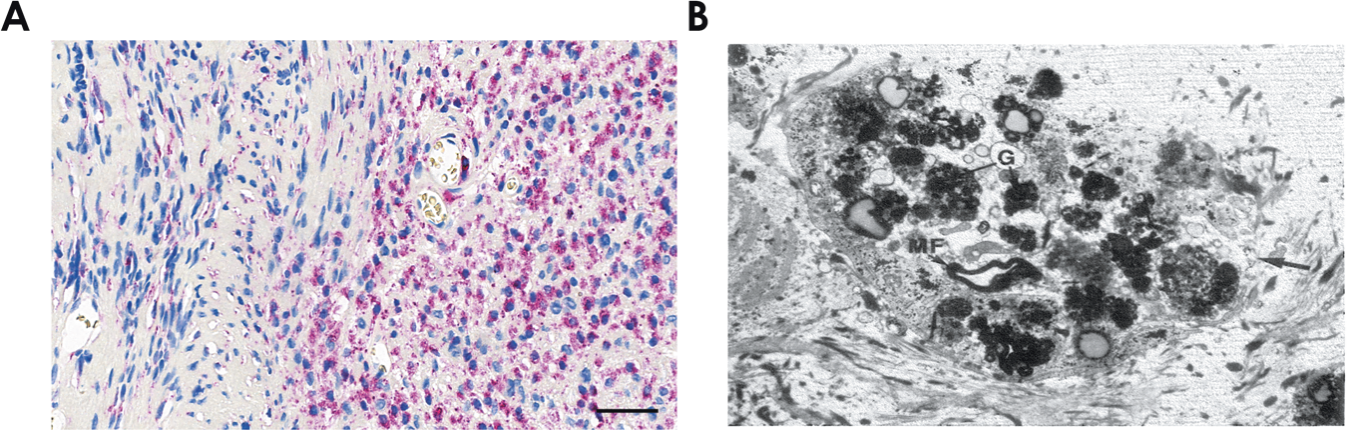
Myelin degradation and phagocytosis are common features of schwannomas. **a** Immunohistochemistry for the lysosomal marker protein CD68 (purple color) in an Antoni A area (left side), Antoni B area (right side), and a transition zone of a schwannoma. Based on the staining pattern, lysosomes appear to be predominantly present in the Antoni B area, in addition to Antoni B-specific tissue features like vacuoles/microcysts and presence of foamy macrophages. Scale bar = 50 µm. **b** Electron micrograph depicting the cytoplasm of a tumor cell inside a schwannoma. A large number of granules (G) can be seen, presumably phagocytic vesicles. Furthermore, a myelin figure (MF) as a sign of myelin phagocytosis and degradation by the tumor cell is present. This image has been reprinted from [8].

**Fig. 4:**
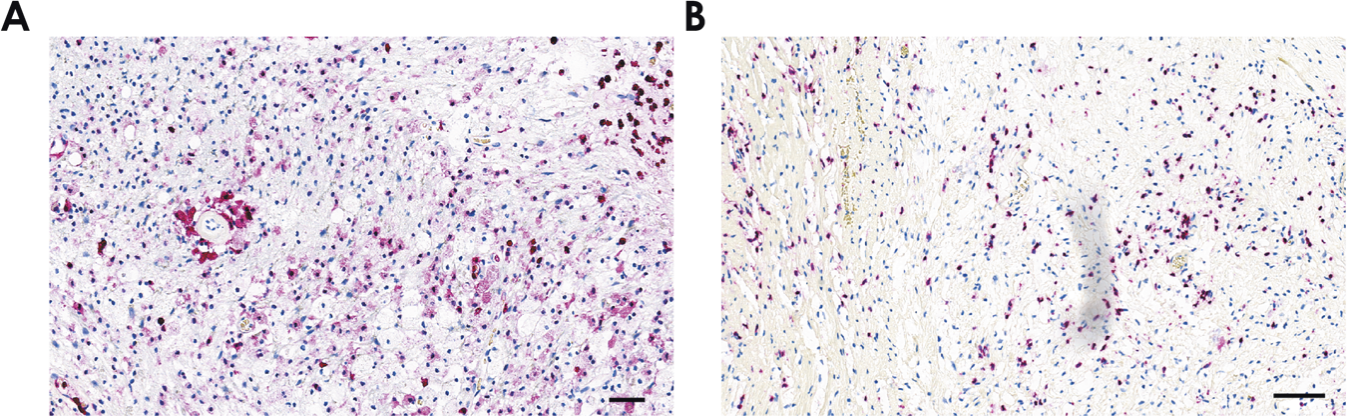
Macrophages and T-cells are part of the schwannoma microenvironment. **a** Immunohistochemistry for the macrophage marker protein MAC387 in a schwannoma. Numerous foamy macrophages are seen within the depicted Antoni B tissue. These are characterized by a large, loosened cytoplasm and small nuclei of different shapes, in contrast to elongated nuclei from tumor cells seen in Antoni A areas. Scale bar = 50 µm. **b** Immunohistochemistry for the T-cell marker protein CD3 in a schwannoma. Heterogenously distributed T cells with small nuclei and cytoplasm are spread throughout this Antoni B tissue. Scale bar = 100 µm.

Further evidence for the transition theory is provided by early experimental findings, showing that Antoni B tissue exhibits many histopathological features reminiscent of Wallerian degeneration following PNS injury [8]. The general stroma is loosened, sometimes even edematous and contains microcystic fields (Fig. 2). Schwann cells and other cell types, primarily inflammatory cells, appear heterogeneously distributed compared with the nuclear rows seen in Antoni A-type tissue and show a loosened and enlarged cytoplasm. Furthermore, they contain a high number of lysosomes and myelin figures, both indicative of active phagocytosis of myelin sheaths by tumorigenic Schwann cells, as demonstrated by electron microscopy and immunohistochemistry for the lysosomal marker protein CD68 (Fig. 3). Macrophage infiltration in Antoni B tissue, in addition to T lymphocytes, further supports the theory of Antoni B areas resembling degenerated Antoni A areas with a tissue degeneration-like process taking place [1] (Fig. 4).

It remains to be elucidated whether a “reciprocal transformation” between Antoni B and Antoni A tissue can occur, thereby generating a cycle of degeneration and formation of new tumor tissue.

## Understanding peripheral nerve de- and regeneration is the key to understanding schwannoma development

### Anatomical distribution of schwannomas points to nerve injuries as a trigger for tumor development

As suggested in the previous section, the progression of an already established schwannoma might depend on the cycling between Antoni A and Antoni B tissue. But how does a new tumor develop in the first place? A recent study from our research group might give some hints about the underlying pathogenesis [2]. Schulz et al. showed that mice bearing a heterozygous *nf2* gene mutation in both the neuronal and the Schwann cell compartment develop schwannomas after a single nerve crush injury [2]. One reason proposed for the development of schwannomas in this mouse model is a failure of Schwann cell re-differentiation into myelinating cells, due to absent signals from *nf2*-deficient neurons. Subsequently, Schwann cells upregulate proliferative signaling pathways, leading to the formation of schwannomas in these mice.

Likewise, the distribution of the most frequent location sites of tumor development provides clinical evidence for this “failure-of-nerve regeneration” theory of schwannoma development. Among the cranial nerves, the vestibular, trigeminal, and hypoglossal nerve are most often affected by schwannomas [9]. Along their anatomical course, all three nerves are located within a confined environment and are therefore amenable to physical stress and injury.

The vestibular nerve, for instance, leaves the brain stem at the cerebellopontine angle as part of the eighth cranial nerve and enters the osseous internal acoustic meatus, being enclosed by bone only [10]. The hypoglossal nerve exits the brain through the bony hypoglossal canal to enter the carotid triangle, where the nerve is in front of the atlas, in close proximity to the pulsating carotid artery, the internal jugular vein, and the vagal nerve; thereafter, the stylohyoid, styloglossus, and hyoglossus muscles, as well as the stylohyoid ligament surround the nerve. Schwannomas of the trigeminal nerve can be classified into four subgroups (A–D) [11, 12], all in close proximity at the skull base.

In the PNS, schwannomas typically arise spinally or paraspinally in close spatial relationship to either the vertebra bones or the vertebral canal. They may also originate from big nerves of the extremities at positions near to bone structures [13]. In the upper extremities, the ulnar nerve is predominantly affected by schwannomas, e.g., behind the medial epicondyle (“funny bone” position). In the lower extremities, the peroneal nerve is often affected by schwannomas in proximity to the head of the fibula bone [10, 14].

In summary, clinical evidence suggests that schwannomas may preferentially appear in locations that are prone to nerve injury by compression or physical trauma (“predilection sites”).

Another line of evidence for the “failure-of-nerve regeneration” theory stems from the observation that loud and chronic noise exposure is a risk factor for development of vestibular schwannoma. Strikingly, this risk increases with the duration of the exposure, suggesting a causal relationship between noise and schwannoma formation [15, 16]. Loud noise exposure represents a widespread cause of sensorineural hearing loss and it has been demonstrated that it not only damages the organ of Corti, but also the cochlear nerve. Mice exposed to experimental loud noise (“acoustic over-exposure”) showed electrophysiological signs of nerve damage with striking dys-myelination of the axons, characterized by thinner myelin sheaths and larger internodal distance [17]. This finding represents a typical feature of altered myelin morphology commonly observed after peripheral nerve injury. Following PNS regeneration, affected axons become myelinated to a lower degree compared with the status before the injury—due to several factors, including insufficient electrical stimulation of re-differentiated Schwann cells during the regeneration process [18].

Conclusively, evidence from both basic research and clinical observation suggests that a variety of nerve-injuring factors and subsequent nerve repair processes might promote schwannoma development.

### Peripheral nerve de- and regeneration involves coordinated action of multiple cell types

Once a nerve has been damaged, the nerve part distal to the injury site begins to degenerate by inducing a Schwann cell de-differentiation program, with subsequent upregulation of inflammatory mediators such as cytokines, lipids, and other molecules. These substances recruit immune cells like macrophages, neutrophils, and T cells to the injury site. This is accompanied by the breakdown and fragmentation of myelin, which is initially cleared by Schwann cells and later, to a more substantial degree, by macrophages [7]. The effectiveness of myelin clearance is critical for successful nerve regeneration in the PNS. In contrast, insufficient central nervous system regeneration is partially attributed to delayed myelin clearance in the context of Wallerian degeneration [19].

Schwann cells utilize autophagy [20] and receptor-mediated phagocytosis [21] to clear myelin from the degenerating distal nerve. The engulfment of myelin components activates signaling pathways in Schwann cells, leading to a so-called “repair cell” mode that is essential for proper nerve regeneration [20, 22]. These repair cells are characterized by the upregulation of several cell adhesion molecules, accompanied by high proliferative and migratory activity. A 2015 landmark paper from the research group of Prof. Allison C. Lloyd showed that the “repair cell” Schwann cells migrate along newly formed blood vessels to their target structure. Endothelial cells, required for the formation of new blood vessels, are attracted and instructed by vascular endothelial growth factor (VEGF) signaling to participate in the process of nerve regeneration [23]. Schwann cells, in turn, later act as guiding structures for regenerating axons [24].

In order to complete successful nerve regeneration, macrophages, along with newly expressed and synthesized axonal proteins, instruct Schwann cells to re-differentiate into myelinating cells [7, 25]. One important axon-derived signal, Neuregulin 1 type III—recognized by Schwann cells through the ErbB2 receptor—instigates signaling cascades leading to differentiation and myelin production of Schwann cells in the regenerating nerve [25]. Another key protein, myelin-associated glycoprotein, is expressed and part of the newly formed myelin sheath. By binding to the Nogo receptor on macrophages, it causes macrophage efflux of the nerve, thereby terminating the inflammatory environment during nerve regeneration [26].

### Merlin loss impairs peripheral nerve regeneration in different mouse models

Genetic deletion of the *nf2* gene has been shown to interfere with nerve regeneration in mice [2, 27–29]. Both Mindos et al. [27] and Truong et al. [28] showed that mice bearing a Schwann cell-specific *nf2* knockout exhibited a dramatic reduction of functional and morphological recovery after experimental sciatic nerve injury. This was reflected by the lower number of myelinated axons and fewer Schwann cell–axon interactions following regeneration. The authors further observed increased proliferative activity in merlin-deficient nerves, as well as an overshooting and sustained presence of macrophages, in comparison to regenerating nerves of wildtype mice. Within 2 months post-injury, a macroscopic nerve swelling was visible.

The authors also noted a marked difference in Schwann cell differentiation compared to wildtype nerves, which was consistent with findings from a study from our research group [2]. Classic protein markers of differentiated and myelinating Schwann cells, such as myelin protein zero (P0) or myelin basic protein, were significantly downregulated distal to the injury side [2, 27]. Conversely, protein markers indicating immature and repair mode Schwann cells, such as p75 or c-Jun [20], were markedly upregulated [2, 27].

While these findings were consistent among different studies [2, 27], the study from our research group aimed to address specifically microenvironmental factors with regards to schwannoma development. Sciatic nerve injury was applied to genetically engineered mice with either Schwann cell-specific *nf2* knockout, or a heterozygous *nf2* knockout in both Schwann cells and adjacent neurons. The latter experimental setup resembles the situation of humans affected by NF2 disease, where one copy of the *Nf2* gene is lost in the entire body due to a germline mutation [30]. In both mouse strains, similar swellings to that of Mindos et al. were detected distal to the injury site. Histopathological analysis revealed the presence of tumorlets and onion bulbs, both indicative of human schwannomas.

These findings highlighted for the first time that cell types of the “tumor microenvironment”, other than Schwann cells, also contribute to schwannoma formation. The concept of the “tumor microenvironment” refers to the cooperative interaction between different cell types that are organized and tightly regulated in a spatiotemporal manner. It further adds to the complexity of biological processes by considering not simply the cell type of interest, but also its metabolically active surroundings. Based on this concept, a purely Schwann cell-focused pathogenesis of schwannoma development may be insufficient, considering the importance of the nerve microenvironment.

Our research group had previously shown that the *nf2* gene product merlin regulates the crosstalk between Schwann cells and adjacent axons via the Neuregulin-ErbB2 signaling pathway [31]. In the PNS, axon-derived Neuregulin 1 is a critical regulator of myelin thickness during development [32]. While Neuregulin 1 becomes dispensable for the maintenance of myelin sheaths after development, it regains importance during nerve repair processes and especially for re-myelination following nerve injury [33, 34].

Existing evidence suggests that a multi-leveled nerve microenvironment needs to be considered in schwannoma biology, as instructive cues from axons, inflammatory signals from macrophages and failed regenerative processes contribute to their formation and progression.

### The schwannoma microenvironment is reminiscent of a chronic wound response

The action of macrophages is crucial for Wallerian degeneration and peripheral nerve regeneration in general. Their presence and activation modulate the cellular behavior of their environment through the release of pro- and anti-inflammatory cytokines. Importantly, after completion of peripheral nerve regeneration, macrophages need to disappear from the nerve tissue. However, two studies in mice on the regeneration of *nf2*-deficient nerves have found an overshooting and sustained presence of macrophages, in comparison with wildtype animals [2, 27].

A retrospective study published in 2014 had already shown an inverse relationship between the intake of the anti-inflammatory drug aspirin and tumor growth in sporadic vestibular schwannomas [35]. The significance of macrophages for schwannoma formation and progression is further underlined by a positive correlation between macrophage infiltration and tumor growth [2, 36–39].

The role of other cell types, such as endothelial cells or T cells, with regard to schwannoma biology is not yet fully understood. It has been shown, however, that T-cell subsets appear in schwannomas in a heterogeneous manner [3]. Furthermore, neovascularization of schwannoma may play an important role regarding the growth rate of schwannomas. de Vries et al. (2012) have shown that, in addition to inflammation, blood vessel density contributes to volume increase in sporadic schwannoma [37]. Therefore, VEGF, which is also produced by schwannoma cells, is an attractive target for therapeutic intervention since it has been shown that its expression correlates with the tumor growth rate [40]. Bevacizumab, a monoclonal antibody against VEGF, has been shown to be effective in preventing tumor progression and is one of the few pharmaceutical treatment options available for the treatment of schwannomas [41–43]. However, the specific role of endothelial cells in regulating the schwannoma microenvironment remains to be determined.

In 1986, Dvorak proposed his influential theory, positing that tumors are wounds that do not heal [44]. Based on our current understanding, this might also apply to schwannomas. Earlier in this review, we presented the hypothesis that the histopathological classification of schwannomas in Antoni A and Antoni B tissue is reminiscent of the processes following nerve injury—including Wallerian degeneration and nerve regeneration. Loss of the *nf2* gene produces a devastating regeneration defect in genetically engineered mice, resulting in a continuous wound response signaling.

Therefore, it is essential to understand how schwannoma tumorigenesis works on a molecular level, thus unraveling the mechanisms of the tumor suppressive functions of the *nf2* gene product merlin. So, which signaling pathways are primarily affected by merlin, which are responsible for tumor cell proliferation, and which are responsible for the dysregulation of the cellular communication in the tumor microenvironment? Finally, which signaling pathways and which cell types need to be targeted therapeutically in order to restrict schwannoma formation and progression?

## Merlin-dependent signaling pathways and potential targets for therapeutic intervention

**Fig. 5:**
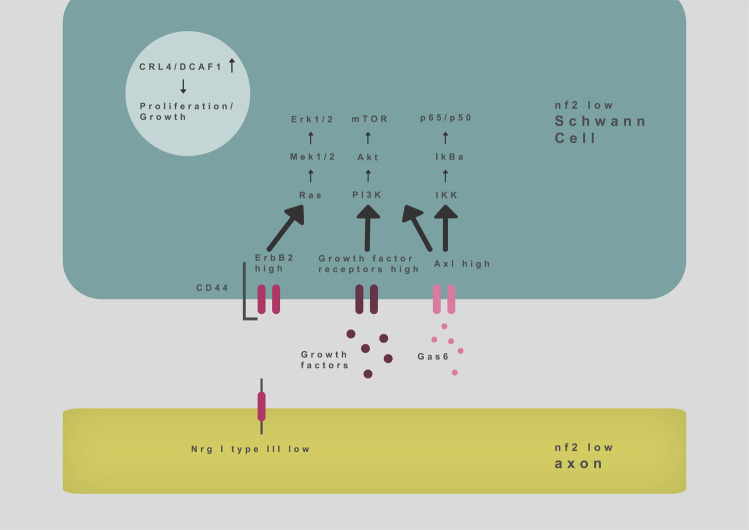
Simplified scheme illustrating major intra- and intercellular signaling pathways implicated in schwannoma growth.

Tremendous efforts have been made in the past two decades to unravel merlin’s cell biological effect on various signaling pathways. These studies have established that merlin exerts its tumor suppressive function in several cellular compartments: (1) at the cell membrane, by regulating growth factor receptor activation [45, 46] and extracellular matrix (ECM) contact-induced intracellular signaling [47]; (2) in the cytoplasm, e.g., by inhibiting signal transduction of the small G proteins Ras and Rac [48], as well as by inhibiting several kinases [46]; (3) in the nucleus, by suppressing the activity of the ubiquitin ligase CRL4^DCAF1^ [49] (Fig. 5).

The cluster of differentiation protein 44 (CD44), which binds hyaluronate of the extracellular matrix, is a well-studied molecule at the cell membrane. CD44-related signaling can serve as a cellular sensor to estimate the cellular density around a cell. When Schwann cells are in a high cell density environment, the protein merlin becomes dephosphorylated and inhibits growth factor-induced signaling pathways, such as the Ras pathway [48, 50]. Referred to as “contact-inhibition of proliferation”, this phenomenon is deactivated in merlin-deficient Schwann cells. Hence, tumorigenic Schwann cells cannot sense the cell number in their environment and maintain proliferation.

Intracellularly, merlin regulates the growth factor receptor-induced activation of the small G proteins Ras and Rac, which in turn activate mitogen-activated protein kinase (MAPK) signaling [48, 51]. Targeting MAPK signaling, e.g., by MEK inhibitors, therefore appears to be another therapeutic option deserving of future investigation, according to several preclinical studies [52, 53].

In addition, merlin-mediated inhibition of mTOR signaling has been shown to contribute to merlin’s tumor suppressive function [46]. It has been proven that mTOR signaling is one of the most important pathways regulating cell proliferation [54]. Allograft experiments in mice revealed that pharmacological inhibition of mTOR signaling could reduce schwannoma growth in vivo [55].

The TAM receptor family is a crucial membrane-bound receptor subgroup that regulates proliferation. Particularly, the AXL receptor is overexpressed and hyperactivated in primary human schwannoma, as well as in merlin-deficient Schwann cells [56]. Ammoun et al. have shown that downstream signaling of AXL is NF-κB-dependent, which in turn is one of the most important signaling pathways for the expression of inflammatory cytokines.

Another important signaling pathway for cellular proliferation is the Hippo pathway. Merlin regulates Hippo signaling on various levels, e.g., by providing a connection between membrane receptors and cytoplasmic effector proteins of this pathway, or by regulating Hippo pathway components through the ubiquitin ligase CRL4^DCAF1^. Activation of merlin stimulates Hippo signaling, resulting in the inhibition of the principal Hippo effector protein, yes-associated protein, a potent oncogene that stimulates cellular growth and survival [57–59].

Furthermore, merlin can regulate the expression of growth factor receptors like the receptor tyrosine kinase ErbB2 [60]. Over-expression of ErbB2 strongly induces growth factor-induced signaling, even in the absence of a ligand [61]. An inhibitor of ErbB2, Lapatinib, has shown promising results in controlling progressive vestibular schwannomas in NF2 patients [62].

Research from our group further revealed that ErbB2 expression in Schwann cells could be upregulated as a consequence of genetic *nf2* deletion in the neuronal compartment of peripheral nerves [31]. We hypothesize that elevated ErbB2 levels on Schwann cells display a compensatory reaction to reduced expression of Neuregulin 1 type III on axons. This prompted us to conduct a preclinical trial (unpublished results) by administering recombinant Neuregulin 1 to schwannoma-bearing mice as a “protein replacement therapy” [63]. In human application, the use of recombinant human Neuregulin 1 is considered clinically safe as a result of clinical trials for heart failure [64, 65].

## Summary

Schwannomas are Schwann cell-derived nerve sheath tumors that appear sporadically and in association with genetic tumor syndromes such as NF2. Countless lines of evidence support the prevailing model that tumorigenic transformation of Schwann cells is caused by loss-of-function mutations of the *Nf2* tumor suppressor gene. However, recent advances in clinical and basic research suggest that a purely Schwann cell-focused pathogenesis of schwannoma tumors may not be sufficient considering the role of the other cell types involved.

This concept, referred to as “tumor microenvironment”, further adds to the complexity of biological processes by including not only the cell type of interest, but also its metabolically active surroundings and their reciprocal interactions. This increasing intricacy, on the other hand, presents more opportunities for the identification of new therapeutic targets and strategies, urgently needed for debilitating tumor entities such as schwannomas.

**Fig. 6:**
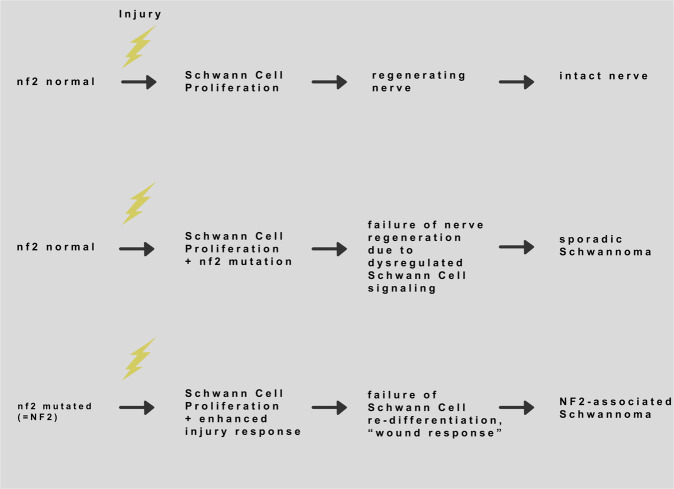
Simplified scheme featuring three possible scenarios that may occur after nerve injury, depending on the underlying genotype regarding the Nf2 gene and DNA damage/mutations that may occur during nerve regeneration.

According to one hypothesis, for which we tried to compile the available evidence in this paper, schwannomas can be regarded as chronic wounds of peripheral nerves. Most Schwann cells are quiescent in a resting non-injured nerve, but de-differentiate and enter proliferation rapidly after a nerve injury [66, 67]. Thus far, several groups have reported on a dramatic nerve regeneration defect in *nf2*-deficient mouse models. One main reason for this severely impaired regeneration seems to be the incapacity of Schwann cells to re-differentiate (Fig. 6). A lack of Schwann cell re-differentiation results in sustained cell proliferation and eventually tumor formation. Importantly, Schwann cell differentiation is not only controlled by Schwann cell-intrinsic programs, but also instructive signals from adjacent axons as well as the presence and composition of an inflammatory milieu.

*Nf2* loss-of-function mutations appear to transform peripheral nerves into a “lesion-proned” state—vulnerable to physical stress and injury. This too could explain distinct clinical findings according to which human NF2 patients, carrying an *Nf2* germline mutation, may accumulate a vast multitude of “microlesions” along their peripheral nerves [68]. It could further help to clarify why certain nerves and localizations appear to be predominantly affected in human patients; in some cases, a close spatial relation of nerve and bone structure seems apparent (so-called “predilection sites”).

In the end, we would like to emphasize: Genes load the gun and the environment pulls the trigger, on multiple levels; first, the anatomical conditions leading to nerve injuries, then the cellular microenvironment—which critically controls the growth behavior of the schwannoma—and lastly, environmental factors, which might also have an influence on a tumor which, according to Dvorak, is a “never healing wound”.
